# Scientific evidence for the management of dentin caries lesions in pediatric dentistry: A systematic review and network meta-analysis

**DOI:** 10.1371/journal.pone.0206296

**Published:** 2018-11-21

**Authors:** Tamara Kerber Tedesco, Thais Gimenez, Isabela Floriano, Anelise Fernandes Montagner, Lucila Basto Camargo, Ana Flávia Bissoto Calvo, Susana Morimoto, Daniela Prócida Raggio

**Affiliations:** 1 Department of Orthodontics and Pediatric Dentistry, School of Dentistry, University of Sao Paulo, Sao Paulo, Sao Paulo, Brazil; 2 Graduation Program, School of Dentistry, Ibirapuera University, Sao Paulo, Sao Paulo, Brazil; 3 School of Dentistry, Paulista University, Campinas, Sao Paulo, Brazil; University of Zurich, SWITZERLAND

## Abstract

**Background:**

A systematic quantitative evaluation of the available evidence of the treatment for caries lesions in primary teeth that considers how different caries progressions lead to the need for distinct interventions might provide additional useful information for clinical evidence-based decision making. The aim of this systematic review and network meta-analysis was to verify the effect of the treatments on caries lesion arrestment (CLA) or the success rate (SR) of dentin caries lesion treatments in the primary teeth.

**Methods:**

A search was conducted using the MEDLINE/PubMed, Web of Science and Scopus databases through December 2017. The primary search terms used in combination were primary teeth, caries lesion and restoration. The grey literature was also screened, as were the reference lists of eligible studies. A search of prospective studies with at least 12 months of follow up that compared different techniques was performed. The exclusion criteria were the absence of a comparison group; no evaluation of different restorative techniques; the evaluation of other outcomes unrelated to this review; and the recruitment of specific patient. The risk of bias was evaluated by the tools: the Cochrane Handbook for Systematic Reviews of Interventions and ROBINS-I. A network meta-analyses and meta-analyses were conducted considering CLA or SR as outcomes according to the surface involved and the depth of progression.

**Results:**

Of the 1671 potentially eligible studies, 15 were included. For occlusal surfaces, only two studies presented data regarding the outer half of the dentin, with conventional restorative treatment (CRT) using composite resin showing superior results; five studies presented data regarding the depth of caries lesions, and CRT with compomer resulted in the best results. Seven studies considered occlusoproximal surfaces, and the Hall technique showed the best SR among the evaluated treatments. Finally, two annual applications of silver diamine fluoride showed the best nonrestorative approach to arrest caries lesions on occlusal and smooth surfaces.

**Discussion/Conclusions:**

The treatments for dentin caries lesions in primary teeth depend on the depth of progression and the surface involved. However, few of the included studies provided evidence to strongly recommend the best treatment option.

**Other:**

Funding: FAPESP; Systematic review registration number—PROSPERO CRD42016037784.

## Introduction

The current scenario in dentistry indicates a high prevalence of dental caries across different age groups and populations [1], despite several existing prevention programs and the global use of fluoride dentifrice [2]. Especially in pediatric dentistry, this result is of great concern because caries is the most important risk factor for developing new caries lesions [3]. Thus, children with an active caries lesion in their primary dentition can also present with caries lesions in their permanent dentition [4].

Despite the knowledge and scientific evidence regarding the prevention of dental caries [5,6], information regarding the effectiveness of different treatment methods proposed for active caries lesions remains lacking. Treatments with strong scientific support have not been identified. A need exists for systematic reviews that compare the several available management options and consider both caries lesion arrestment and treatment success as outcomes.

Previous systematic reviews that evaluated the preferred treatment for dentin caries lesions in primary teeth have focused on comparing only two types of treatments or the same treatment with different restorative materials, considering only the type of surface involved (occlusal or occlusoproximal) [7–9] or even other outcomes such as the prevention of secondary caries lesion [10,11]. The gap in the evidence that considers lesions of different depths and the number of surfaces involved that affect treatment effectiveness makes recommending the best treatment for dentin caries lesions with different levels of progression challenging.

Thus, a systematic quantitative evaluation of the available evidence on the treatment for caries lesions in primary teeth that considers how different caries progressions lead to the need for distinct interventions might provide more useful information for clinical evidence-based decision making. Therefore, this systematic review and network meta-analysis compared the performance of the available treatments for dentin caries lesions, regardless of nearness to pulp or pulp involvement in primary teeth, on caries lesion arrestment (CLA) or the success rate (SR) and considered the different progression depths and surfaces involved.

## Material and methods

This systematic review and network meta-analysis was reported according to the PRISMA-NMA extension [12] (S1 Table) and was registered at the International Prospective Register of Systematic Reviews (PROSPERO) (protocol number #CRD42016037784; available from: http://www.crd.york.ac.uk/PROSPERO/display_record.php?ID=CRD42016037784).

### Search strategy

A systematic search of the available studies in the literature was conducted using the following electronic databases: MEDLINE/PubMed, Web of Science and Scopus. The grey literature (OpenSigle/Opengrey) and the reference lists of identified full texts were also screened to retrieve additional relevant studies that might fulfill the inclusion criteria. No restriction was placed on the language or year of publication. The last search was performed on December 14, 2017.

A search strategy was developed for the MEDLINE/PubMed database and then adapted for the others based on the following PICO question: "Which is the best treatment for CLA or SR of the dentin caries lesions of primary teeth?" The results of the different databases were cross-checked manually to locate and eliminate duplicate studies. The complete search strategy is shown below: (amalgam OR resin* OR composite* OR composite resin* OR resin composite* OR compomer* OR polyacid modified composite resin* OR polyacid-modified composite resin* OR ultra-conservative treatment OR ultraconservative restorative treatment OR UCT OR dental restoration* OR restoration OR dental restoration, permanent OR tooth restoration OR teeth restoration OR glass ionomer cement* OR glass-ionomer cement* OR GIC OR ART OR atraumatic restorative treatment OR atraumatic restorative procedure) AND (caries OR carious OR tooth decay OR teeth decay OR dental caries OR cavitated caries lesion OR cavitated carious lesion) AND (longevity OR survival OR success OR caries lesion progression OR caries lesions progression OR caries arrestment) AND (primary teeth OR primary tooth OR deciduous teeth OR deciduous tooth OR deciduous OR primary dentition OR teeth, deciduous OR tooth, deciduous).

### Selection criteria

Initially, the titles and abstracts of the potentially eligible studies identified using the databases were evaluated by two independent reviewers (TKT and TG), who were previously trained and calibrated for study selection (Kappa = 0.8). The studies were considered eligible if they were prospective clinical studies with at least 12 months of follow up evaluating treatments for dentin caries lesions without a possible nearness to a pulp or pulp involvement in primary teeth. Studies without listed available abstracts were fully assessed for full copy inclusion/evaluation.

After the first evaluation, the studies that meet the inclusion criteria had their full texts independently reviewed; then, those with at least one exclusion criterion were considered as ineligible. The exclusion criteria were the absence of a comparison group; no evaluation of different restorative techniques; the evaluation of other outcomes unrelated to this review; and the recruitment of specific patient groups (e.g., patients receiving medication or those with special needs). When more than one study included the same sample, the one that presented more complete data was considered. For data synthesis, we only included the data from manuscripts that presented separate data based on surface involved, depth of progression, or both.

All available approaches to treat dentin caries lesion of primary teeth was considered in this systematic review. Atraumatic restorative treatment (ART) was considered as a restorative procedure that included caries removal using only hand instruments (i.e., spoon excavators) and restoration with high-viscous glass ionomer cement (HV) without the use of a rubber dam. Alternatively, conventional restorative technique (CRT) was considered as including caries removal using rotary instruments and restoration with any restorative material, including the use of a rubber dam. Thus, studies reporting treatment procedures that differed from those definitions were not included in the present review.

All discrepancies regarding the eligibility criteria were resolved in consultation with a third reviewer (DPR), and consensus was reached.

### Data collection

Studies that fulfilled the eligibility criteria were considered for further assessment of risk of bias and data extraction. Data were extracted from the full texts of the included articles using a standardized form. The authors categorized similar information into groups according to the main outcomes of interest. One of the reviewers (TKT) collected the required information from full-text eligible studies, and a second reviewer (TG) independently checked all of the collected data using those standardized outlines.

For each included study, the following data were systematically extracted: publication details (authors and year), sample characteristics (number and age of participants, number of treatments performed per group, caries experience, level of progression [surfaces involved and depth of progression], and dropouts), study methodology (design, setting, training of operators, blinding of examiners and index used to evaluate the outcome) and outcome information (follow-up time and success rate). The success rate was considered as the survival of treatment (no need of repair) or the success of treatment (treatment appears satisfactory, no clinical signs or symptoms of pulpal pathology or caries arrested).

For studies that did not report this precise and required information, the authors of these studies were contacted via email to provide missing data or more information. Only the data of interest within the selected studies were extracted for analysis.

### Assessment of risk of bias

After data collection, two independent reviewers (TKT and IF) assessed the potential risk of bias of the included studies. A specific protocol developed to analyze intervention studies was used to assess the randomized clinical trials included: the Cochrane Handbook for Systematic Reviews of Interventions 5.0.1 [13]. This tool is divided into six major questions regarding randomization, blinding, analysis of the outcome data and a possible imbalance in the baseline sample characteristics.

The risk of bias assessment of observational study was evaluated according to The Risk of bias in non-randomized studies of interventions (ROBINS-I). This tool is based on seven domains that included bias due to confounding, selection of participants, classification of interventions, deviations from intended interventions, missing data, measurement of outcomes and selection of the reported result [14].

Both risk of bias assessment tools require that researchers rank the item as "yes" (low risk of bias), "no" (high risk of bias) or "unclear" (unable to identify information or uncertainty about potential bias) for each included study. For the final risk of bias classification, disagreements between the reviewers were resolved via consensus.

### Evaluation of quality of evidence—GRADE approach

The GRADE tool was used to evaluate the certainty of the evidence as a strategy to consider the confidence in the results from a meta-analysis or network meta-analysis. For this procedure, the adaptation of Salanti [15] was used to evaluate the confidence in treatment ranking estimated by the network meta-analysis across five domains: study limitations, indirectness, inconsistency, imprecision and publication bias.

Confidence is scored as very low, low, moderate and high, and the reason for downgrading is reported.

### Data synthesis and statistical methods for the network meta-analysis and meta-analysis

Direct evidence was computed using a random-effects model meta-analysis in which two treatments (experimental treatment [sealing] vs. control treatment [conventional restorative treatment with resin composite]) were evaluated for the same level of caries lesion progression. Heterogeneity was evaluated using the I^2^ test.

Otherwise, when more than two treatments were considered across different studies with regard to the same depth or surface involved, a network meta-analysis was conducted that synthesized direct and indirect comparisons to strengthen the evidence. Indirect comparisons were performed according to the method proposed by Bucher et al. [16]. To simultaneously consider both direct and indirect evidence, we performed a Bayesian mixed-treatment comparison (MTC). Because all the treatment options were performed on similar groups of patients, this network meets the transitivity assumption. First, we conducted MTC analyses using fixed- and random-effects models. The goodness-of-fit of the models was measured using the residual deviance and deviance information criteria (DIC). Because the DIC value of random-effects model was lower, we used the MTC random-effects model with homogeneous between-trial variability. A node split analysis for inconsistency was not performed because of the insufficient amount of data. The network meta-analysis and meta-analysis were conducted using the R package “stats”, version 2.15.3 (R Core Team, 2012, Vienna, AUT).

For each included study, the risk ratio and 95% confidence intervals were calculated. Studies with multiple arms need specific care to avoid double counting, one of the possibilities of approach is to perform a mixed treatment comparison (MTC), allowing each two groups to be compared indirectly through comparisons with the third [13], as we did. The Becker-Balagtas method was used to calculate log RRs to accommodate data pooling from split-mouth (cluster) and parallel-group studies in a single meta-analysis and facilitate data synthesis [17,18].

## Results

### Study selection

**Fig 1:**
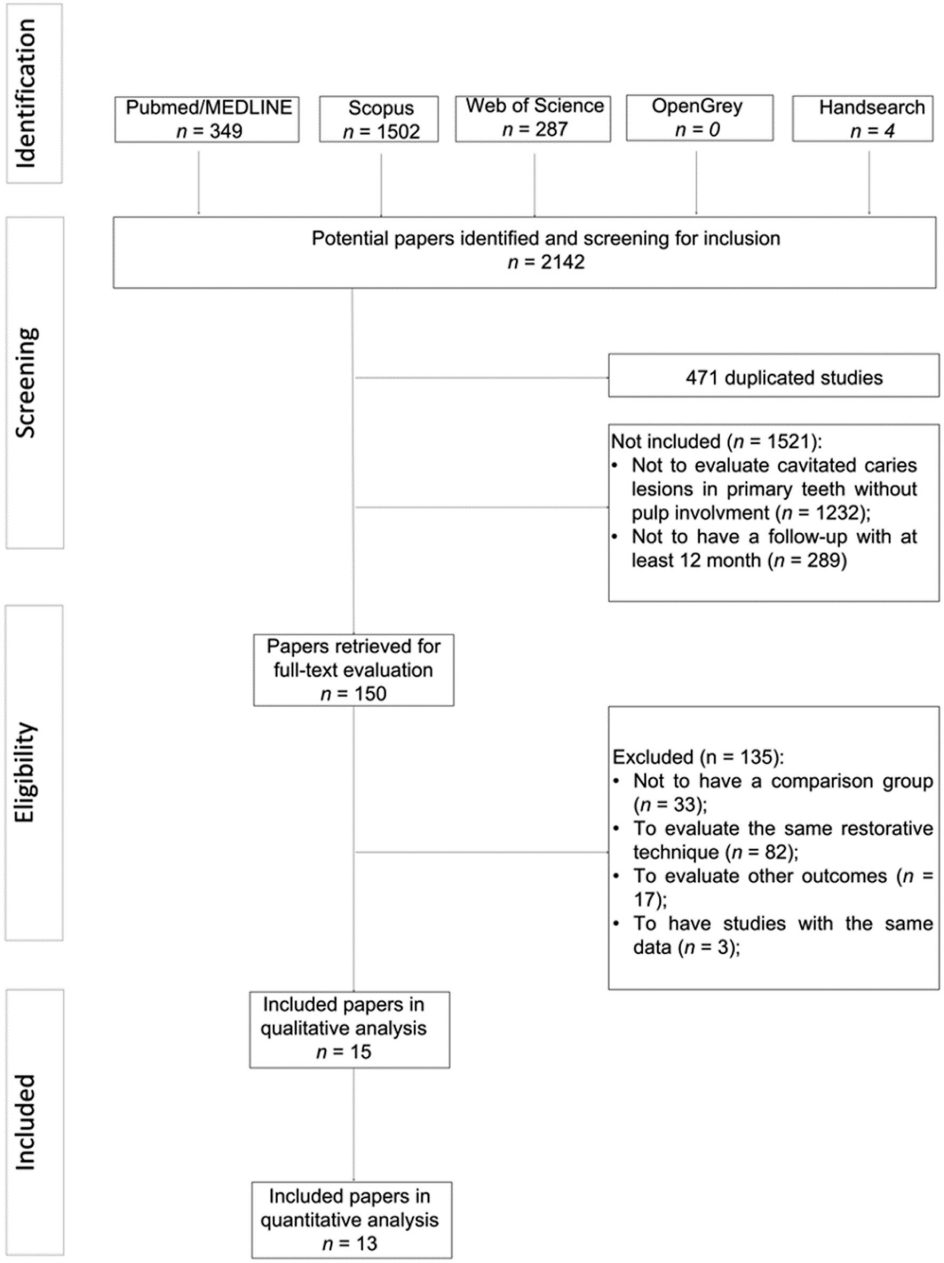
Flowchart of the manuscript-screening process and their inclusion in the systematic review and statistical analysis.

The systematic literature search identified 2142 potentially relevant manuscripts, and four additional were retrieved from a manual search of the reference lists of eligible manuscripts. A total of 471 titles were considered as duplicates and were therefore excluded. No eligible manuscripts were found in the grey literature. After the title and abstract screening, 1521 manuscripts were considered ineligible, mainly for not being related to the scope of this review (81%). A total of 150 remaining studies were retrieved as full-text manuscripts for more detailed information, and 135 articles excluded. The main reason for exclusion was that these studies evaluate the same restorative technique (61%; S2 Table). Finally, fifteen publications fulfilled the eligibility criteria and were included in the current systematic review. Thirteen presented appropriate data and were included in meta-analysis or network meta-analysis. Fig 1 displays the details of the systematic search and the study selection process.

### Study characteristics

The major characteristics of the included studies are summarized in Tables 1 and 2. Most of the studies were randomized clinical trials with parallel groups (60%) [19–27]. Only one observational study was included [28]. School was the location of choice for performing the treatments according to seven papers (46.7%) [19,21,22,25–27,29]. Ten studies reported data regarding the success of the treatments onto occlusoproximal surfaces [19–23,28–32]. Furthermore, the majority of manuscripts performed CRT (80%) to treat dentin caries lesions [19–24,28–33]. Only one study evaluated nonrestorative caries treatment (NRCT) [23] and ultraconservative caries treatment (UCT) [22]. The ART criteria were the most commonly used outcome evaluation indices (33.3%) [19–21,30,31]. In addition, the most frequently reported follow-up period was one year (73.3%) [19,22–27,29–31,33].

**Table 1:** Main characteristics of included studies.

Author/Year and Country	Design	Setting	n (patient)	Age (years)	Caries experience	Surfaces involved and deep of progression	N in according to the treatment
**Louw et al., 2002 (South Africa)**	RCT—Parallel Groups	School	401	6–9	> 60%	Occlusal Occlusoproximal	(O) ART– 186 (O) CRT—211 (OP) ART– 261 (OP) CRT– 177
**Taifour et al., 2002 (Syria)**	RCT—Parallel Groups	Clinic	835	6–7	ceo-d = 4.4	Occlusal Occlusoproximal	(0) ART– 476 (O) CRT– 380 (OP) ART– 610 (OP) CRT—425
**Honkala et al., 2003 (Kuwait)**	RCT—Split-mouth	Clinic	42	2–9	At least two cavitated dentin caries lesions	Occlusal Occlusoproximal	(0) ART– 26 (O) CRT– 26 (OP) ART– 9 (OP) CRT—9
**Yu et al., 2004 (China)**	RCT—Split-mouth	Clinic	60	7.4 ± 1.24	At least two cavitated dentin caries lesions	Occlusal Occlusoproximal	(0) ART– 37 (O) CRT AM– 32 (O) CRT HVGIC—45 (OP) ART– 35 (OP) CRT HVGIC -18
**van den Dungen et al., 2004 (Indonesia)**	RCT—Parallel Groups	School	393	6.5 ± 0.50	At least one OP cavitated dentin caries lesions	Occlusoproximal	ART ≈ 200 CRT ≈ 200
**Roberts et al., 2005 (United Kingdom)**	Practice-based research	Clinic	n/m	CRT—7.48 SSC—6.29	At least one cavitated dentin caries lesions	Occlusoproximal	CRT– 1088 SSC—1107
**Ersin et al., 2006 (Turkey)**	RCT—Split-mouth	School	219	6–10	dmft = 5.1	Occlusal Occlusoproximal	(O) ART– 119 (O) CRT– 111 (OP) ART– 96 (OP) CRT—93
**Innes et al., 2011 (Scotland)**	RCT—Split-mouth	Clinic	132	3–10	dmft = 2.5	Occlusal Occlusoproximal	CRT– 132 HT -132
**Mijan et al., 2014 (Brazil)**	RCT—Parallel Groups	School	302	6–7	At least two cavitated dentin caries lesions	Occlusoproximal	(OP) ART– 188 (OP) CRT– 238 (OP) UCT– 219
**Santamaria et al., 2014 (Germany)**	RCT—Parallel Groups	Clinic	169	3–8	dmft = 5.6	Occlusoproximal	CRT– 65 HT– 52 NRCT—52
**Borges et al., 2012 (Brazil)**	RCT—Split-mouth	Clinic	30	5–9	dmft = 2.3	Occlusal—outer half of dentin	Sealing– 30 CRT– 30
**Hesse et al., 2014 (Brazil)**	RCT—Parallel Groups	Clinic	36	4–9	dmft = 6.0	Occlusal—outer half of dentin	Sealing– 17 CRT– 19
**Santos et al., 2012 (Brazil)**	RCT—Parallel Groups	School	91	5–6	DMFT = 3.8	Occlusal and smooth surface	IRT –162 SDF 1x-183
**Zhi et al., 2012 (China)**	RCT—Parallel Groups	School	212	3.8	dmft = 5.1	Occlusal and smooth surface	SDF 1x—218 SDF 2x—239 LVGIC—262
**Duangthip et al., 2016 (China)**	RCT—Parallel Groups	School	304	3–4	dmft = 4.4	Occlusal and smooth surface	SDF 1x—581 SDF 3x—488 NaF 3x—601

Abbreviations: RCT: Randomized Clinical Trial; OP: occlusoproximal; O: occlusal; ART: Atraumatic restorative treatment; CRT: Conventional restorative treatment; SSC: Stainless steel crown; NRCT: Nonrestorative caries treatment; UCT: Ultraconservative treatment; HT: Hall technique; IRT: Interim restorative treatment; SDF: Silver diamine fluoride; LVGIC: Low-viscosity glass ionomer cement; NaF: Sodium fluoride; RS: resin sealant; HVGIC: High-viscosity glass ionomer cement; RC: Resin composite; AM: Amalgam; RMGIC: Resin-modified glass ionomer cement; USPHS: US Public Health Service criteria; PUFA: Index of clinical consequences of untreated dental caries; n/m: Not mentioned.

**Table 2:** Main characteristics of included studies.

Author/Year and Country	Evaluation criteria	Operators -Training	Examiners—blinding	Follow-up	Drop-out (%)	Success rate or Caries lesion arrestment (%)		
**Louw et al., 2002 (South Africa)**	ART criteria	5 trained dentists—n/m	2 —n/m	1 year	19.9	ART (HVGIC and Compomer) HVGIC (O): 96 (OP): 73 Compomer: (O): 98 (OP): 78		CRT (HVGIC e Compomer) HVGIC (O): 94 (OP): 78 Compomer: (O): 98 (OP): 79
**Taifour et al., 2002 (Syria)**	ART criteria	8 dentists—three days of training	3 —n/m	3 years	22.1	ART (HVGIC) (O): 86.1 (OP): 48.7		CRT (AM) (O): 79.6 (OP): 42.9
**Honkala et al., 2003 (Kuwait)**	ART criteria and USPHS	2 dentists—training with expert	2 –blinding not possible	1 year 2 years	17	ART (HVGIC) 1 year (O): 100 (OP): 100 2 years (O): 92.3 (OP): 88.9		CRT (AM) 1 year (O): 100 (OP): 100 2 years (O): 92 (OP): 100
**Yu et al., 2004 (China)**	ART criteria	2 experienced dentists—n/m	2 —n/m	6 months 1 year 2 years	42	ART (HVGIC) 6 months (O): 100 (OP): 82.8 1 year (O): 94.3 (OP): 65.3 2 years (O): 91.4 (OP): 52	CRT (AM) 6 months (O): 100 1 year (O): 100 2 years (O): 88.9	CRT (HVGIC) 6 months (O): 97.8 (OP): 100 1 year (O): 89.8 (OP): 100 2 years (O): 89.8 (OP): 84.6
**van den Dungen et al., 2004 (Indonesia)**	ART criteria	2 dentists and 2 undergraduation student—trained for 1 week	10 trained and calibrated undergraduation students—blind	3 years	41.7	ART (HVGIC) 31.0		CRT (HVGIC) 33.6
**Roberts et al., 2005 (United Kingdom)**	In according to the dentist’s assessment	1 dentist—n/m	1 –not blind (same operator)	Until 7 years	10.2	CRT (RMGIC) 97.3		SSC 97.0
**Ersin et al., 2006 (Turkey)**	USPHS Ryge criteria	3 dentists—n/m	2 trained and calibrated—blind	6 months 1 year 2 years	17.8	ART (HVGIC) 6 months (O): 100 (OP): 90.2 1 year (O): 100 (OP): 83.1 2 years (O): 96.7 (OP): 76.1		CRT (RC) 6 months (O): 100 (OP): 92.3 1 year (O): 98 (OP): 87.5 2 years (O): 91 (OP): 82
**Innes et al., 2011 (Scotland)**	Innes et al., (2007) criteria	17 trained dentists—n/m	17 dentists—not blind (same operator)	Until 5 years	31	CRT (Usual treatment) 52		HT 92
**Mijan et al., 2014 (Brazil)**	PUFA index	3 trained pediatric dentists—n/m	2 trained and calibrated pediatric dentists—n/m	6 months 1 year 2 years 3 years 3.5 years	12.2	ART (HVGIC) 6 months (OP): 100 1 year (OP): 97.7 2 years (OP): 93.1 3 years (OP): 93.4 3.5 years (OP): 88.0	CRT (AM) 6 months (OP): 99.2 1 year (OP): 96.9 2 years (OP): 92.4 3 years (OP): 91.2 3.5 years (OP): 89.0	UCT 6 months (OP): 100 1 year (OP): 95.7 2 years (OP): 88.0 3 years (OP): 88.0 3.5 years (OP): 88.0
**Santamaria et al., 2014 (Germany)**	Innes et al., (2007) criteria	12 trained dentists—n/m	2 trained experienced pediatric dentists—n/m	1 year	12.4	CRT (Compomer) 71	HT 98	NRCT 75
**Borges et al., 2012 (Brazil)**	Criteria reported by Aguilar et al., (2007)	1 trained and experienced dentists—n/m	1 experienced and calibrated—n/m	1 year	13.3	Sealing (RS) 91.6		CRT (RC) 100
**Hesse et al., 2014 (Brazil)**	Criteria reported by Houpt et al., (1983)	1 trained undergraduation student– 1 week of training in patients	1 trained—n/m	6 months 1 year 1.5 years	5.6	Sealing (RS) 6 months: 87.5 1 year: 75.0 1.5 years: 64.7		CRT (RC) 6 months: 100 1 year: 100 1.5 years: 100
**Santos et al., 2012 (Brazil)**	Based on Miller (1959) and Kidd (2010)	1 trained dentist—n/m	1—blinding not possible	6 months 1 year	6.7	IRT (HVGIC) 6 months: 53.1 1 year: 38.6		SDF (30%) 6 months: 84.7 1 year: 66.9
**Zhi et al., 2012 (China)**	Visual and tactile characteristics of caries lesion arrestment	2 trained dentists—n/m	1—blind	6 months 1 year 1.55 years 2 years	15	SDF 1x (38%) 6 months: 31.5 1 year: 37.0 1.5 years: 77.2 2 years: 79.2	SDF 2x (38%) 6 months: 43.3 1 year: 53.0 1.5 years: 82.9 2 years: 90.7	LVGIC 6 months: 31.3 1 years: 28.6 1.5 years: 73.1 2 years: 81.8
**Duangthip et al., 2016 (China)**	Visual and tactile characteristics of caries lesion arrestment	1 dentist—n/m	1—blind	6 months 1 year 1.5 years	9.0	SDF 1x (30%) 6 months: 18 1 year: 20 1.5 years: 40	SDF 3x (30%) 6 months: 31 1 year: 28 1.5 years: 35	NaF 5% 6 months: 10 1 year: 13 1.5 years: 27

Abbreviations: RCT: Randomized Clinical Trial; OP: occlusoproximal; O: occlusal; ART: Atraumatic restorative treatment; CRT: Conventional restorative treatment; SSC: Stainless steel crown; NRCT: Nonrestorative caries treatment; UCT: Ultraconservative treatment; HT: Hall technique; IRT: Interim restorative treatment; SDF: Silver diamine fluoride; LVGIC: Low-viscosity glass ionomer cement; NaF: Sodium fluoride; RS: resin sealant; HVGIC: High-viscosity glass ionomer cement; RC: Resin composite; AM: Amalgam; RMGIC: Resin-modified glass ionomer cement; USPHS: US Public Health Service criteria; PUFA: Index of clinical consequences of untreated dental caries; n/m: Not mentioned.

### Assessment of risk of bias

Details of the assessment of the risk of bias for the clinical trials and practice-based research are displayed in Tables 3 and 4, respectively. Most of the studies were scored as having weak evidence because they did not provide most of the information required.

For clinical trials, all studies were classified as *unclear* using at least two questions because of the lack of information in the manuscripts. On the other hand, all reported the method of random sequence generation, whereas only three studies discussed allocation concealment [12,32,33]. Information about the blinding of participants and personnel and/or outcome assessment was mentioned in four studies [21,26,27,29]. Duangthip et al. [27] was the only study that was deemed to have a low risk of bias with regard to both of these criteria, as well as with regard to the *free of incomplete outcome data* item. Regarding the question about *free baseline imbalance*, two studies reported this information and were indicated as having a low risk of bias [26,27] (Table 3).

**Table 3:** Assessment of risk of bias for the clinical trials included.

	Questions to be considered						
Study	Random sequence generation	Allocation concealment	Blinding of participants and personnel	Blinding of outcome assessment	Free of incomplete outcome data	Free from baseline imbalance	Other sources of bias
Louw et al. (2002)	YES	UNCLEAR	NO	UNCLEAR	UNCLEAR	UNCLEAR	UNCLEAR
Taifour et al. (2002)	YES	UNCLEAR	UNCLEAR	UNCLEAR	UNCLEAR	UNCLEAR	UNCLEAR
Honkala et al. (2003)	YES	UNCLEAR	NO	NO	UNCLEAR	UNCLEAR	UNCLEAR
Yu et al. (2004)	YES	UNCLEAR	UNCLEAR	UNCLEAR	UNCLEAR	UNCLEAR	UNCLEAR
van den Dungen et al. (2004)	YES	UNCLEAR	NO	YES	UNCLEAR	UNCLEAR	UNCLEAR
Ersin et al. (2006)	YES	UNCLEAR	UNCLEAR	YES	UNCLEAR	UNCLEAR	UNCLEAR
Innes et al. (2011)	YES	YES	NO	NO	UNCLEAR	UNCLEAR	UNCLEAR
Mijan et al. (2014)	YES	NO	NO	NO	UNCLEAR	UNCLEAR	UNCLEAR
Santamaria et al. (2014)	YES	YES	NO	NO	UNCLEAR	UNCLEAR	UNCLEAR
Borges et al. (2012)	YES	YES	UNCLEAR	UNCLEAR	UNCLEAR	UNCLEAR	UNCLEAR
Hesse et al. (2014)	YES	UNCLEAR	UNCLEAR	UNCLEAR	UNCLEAR	UNCLEAR	UNCLEAR
Santos et al. (2012)	YES	UNCLEAR	NO	NO	UNCLEAR	UNCLEAR	UNCLEAR
Zhi et al. (2012)	YES	UNCLEAR	UNCLEAR	YES	UNCLEAR	YES	UNCLEAR
Duangthip et al. (2016)	YES	UNCLEAR	YES	YES	YES	YES	UNCLEAR

Concerning the observational study [28], the items about bias due to confounding, in selection of participants into the study, in classification of interventions, and due to deviations from intended intervention was scored as *yes* and were considered as having a low risk of bias. However, the items about the bias due to missing data, in measurement of outcomes and in the selection of the reported were categorized as *unclear* because of our inability to identify this information in the manuscript. A high risk of bias was identified only with regard to the item addressing statistical analysis (Table 4).

**Table 4:** Assessment of risk of bias for observational study included.

Manuscript Check-list for Non-randomized studies of intervention	Roberts et al., 2005
Bias due to confounding	Yes
Bias in selection of participants into the study	Yes
Bias in classification of interventions	Yes
Bias due to deviations from intended intervention	Yes
Bias due to missing data	UNCLEAR
Bias in measurement of outcomes	UNCLEAR
Bias in selection of the reported result	UNCLEAR
Was appropriate statistical analysis used?	No

### Evaluation of quality of evidence—GRADE approach

The evaluation of the quality of evidence is displayed in Table 5. Study limitations were the most common reasons for downgrading the confidence of the analysis. Indirectness and intransitivity were assumed based on the similarity of the patients, treatments and outcomes across included studies. Inconsistency was considered adequate because the studies included in the analysis were homogeneous. Furthermore, no inconsistency was verified in the network meta-analysis. The ranking of treatments in the network meta-analysis showed high precision. However, publication bias was not considered because of the small number of studies included in each analysis. The confidence of the results was low for the outer half of the occlusal, occlusoproximal and occlusal/smooth surfaces.

**Table 5:** Evaluation of quality of evidence—GRADE approach.

GRADE Domain Meta-analysis	Confidence	Reason to downgrading
Outer half of occlusal surface	Low	Study limitation—High risk of bias
Occlusal surface	Low	Study limitation—High risk of bias
Occlusoproximal surface	Low	Study limitation—High risk of bias
Occlusal and Smooth surface	Moderate	Study limitation—Moderate risk of bias

### Network meta-analysis and meta-analysis

The analyses were performed depending on the surface involved in the dentin caries lesion, the depth of progression, or both.

**Fig 2:**
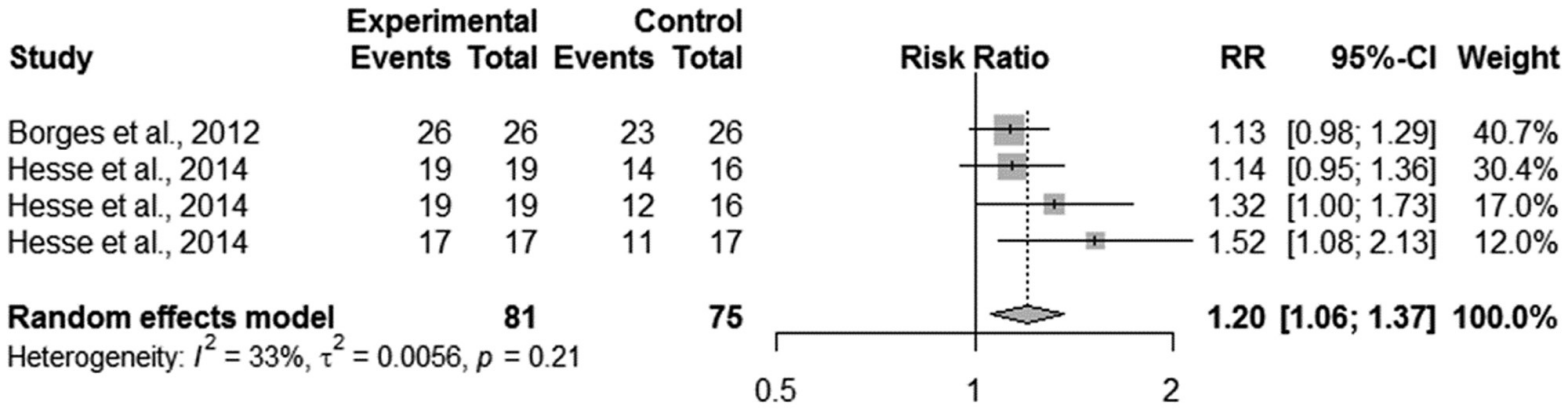
Random-effects meta-analysis evaluation of the success rate of restorative treatments in outer half of dentin on occlusal surface—Experimental treatment (sealing) vs. Control treatment (Conventional restorative treatment with resin composite).

Initially, the data were synthesized across studies that evaluated dentin caries lesions in the outer half of the dentin on the occlusal surface. A meta-analysis was performed since only two restorative treatments were considered. Heterogeneity among included studies was not observed (p = 0.9567; I^2^ test = 0%). The primary outcome of this comparison was the success rate. Considering the overall analysis of the included clinical trials with regard to the success rate outcome, resin composite (RC) showed a higher success rate than sealing with a resin sealant (RR = 11.16, 95% CI: 2.46–50.62) (Fig 2).

**Fig 3:**
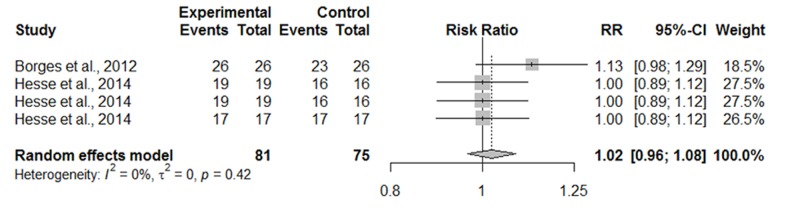
Random-effects meta-analysis evaluation of the caries arrestment of restorative treatments in outer half of dentin on occlusal surface—Experimental treatment (sealing) vs. Control treatment (Conventional restorative treatment with resin composite).

However, when caries arrestment was considered as the primary outcome, no difference was observed between the restorative treatments (RR = 7.89, 95% CI: 0.39–160.91) (Fig 3).

**Fig 4:**
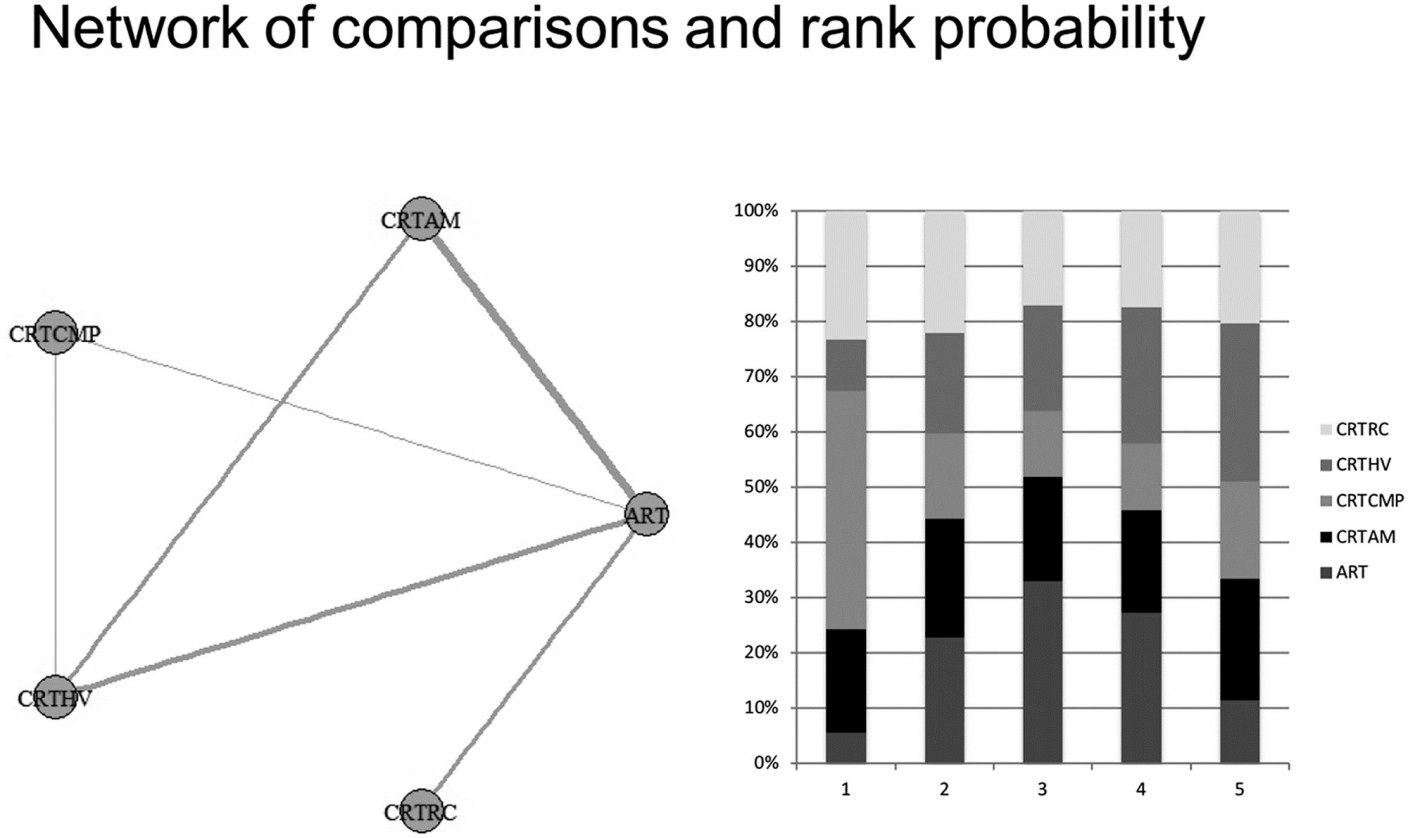
Network meta-analysis of the success rate of restorative treatments in dentin caries lesion on occlusal surface—Geometry of the network and probability ranking of the best behaviour of the treatments on occlusal surface.

For the studies that considered only the occlusal surface without information about the depth of progression, a network meta-analysis was conducted, and five studies that considered five treatment options were included. Heterogeneity was not observed among the included studies. The primary outcome of this comparison was the success rate (S3 Table). The results of the direct and indirect comparisons to provide the network meta-analysis was displayed in S1 and S2 Figs, respectively. The rank probability showed that the best results for occlusal surfaces are expected using CRT with compomer (CRT CMP) (1°). After that, the final ranking was ART (2°), CRT HV (3°), CRT with amalgam (CRT AMG) (4°) and CRT with resin composite (CRT RC) (5°) (Fig 4).

Seven studies were considered for the analysis that considered dentin caries lesions on occlusoproximal surfaces, without information about the depth of progression, and eight possible treatments were evaluated. One manuscript evaluated restorative techniques that were not addressed in the others studies; thus, the data from this observational study were not included in the network meta-analyses.

**Fig 5:**
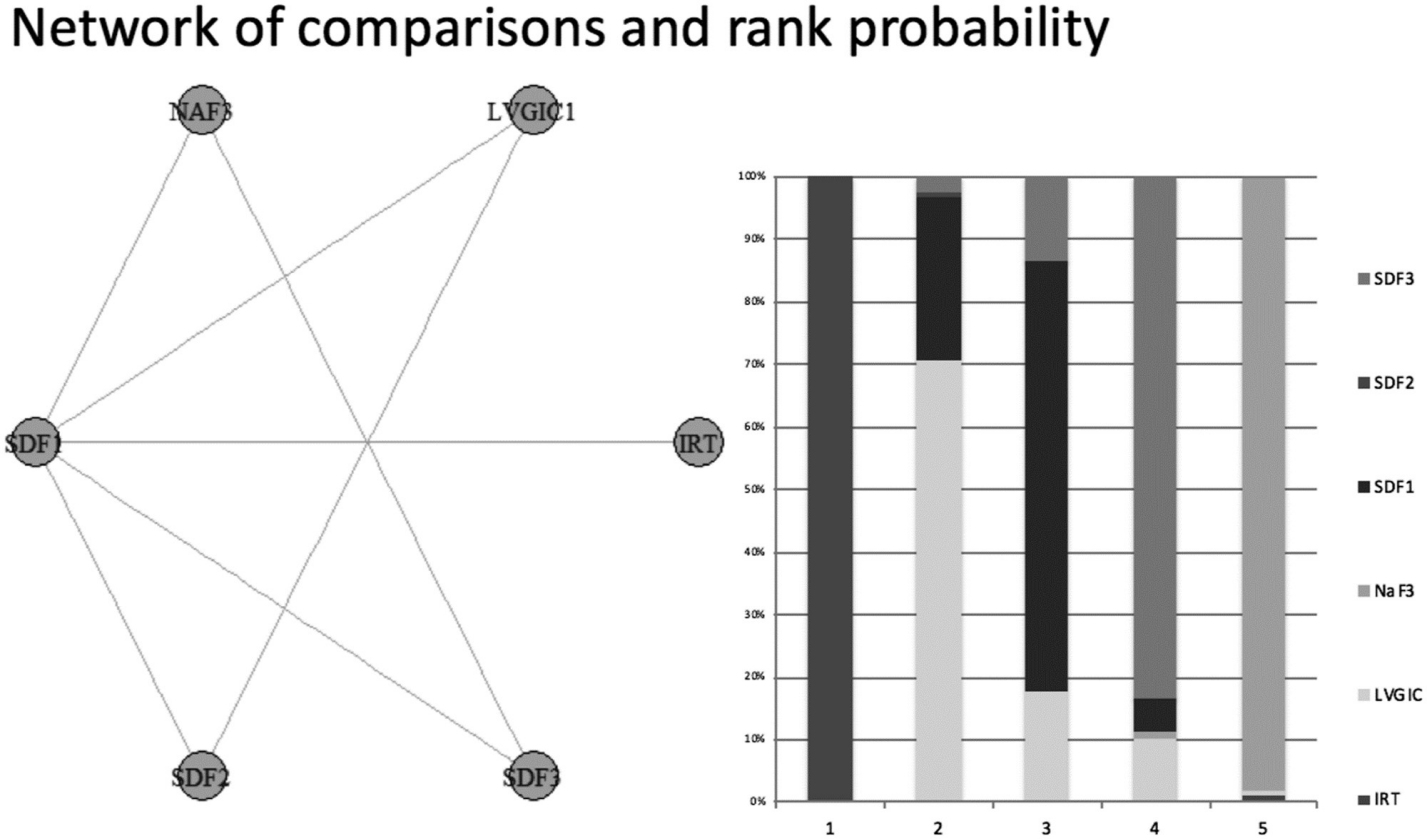
Network meta-analysis of the success rate of treatments options in dentin caries lesion on occlusoproximal surface—Geometry of the network and probability ranking of the best behaviour of the treatments on occlusoproximal surface.

Thus, a network meta-analysis was conducted. The primary outcome of this comparison was the success rate (S4 Table). The results of the direct and indirect comparisons to provide the network meta-analysis was displayed in S3 and S4 Figs, respectively. Heterogeneity was not observed among the included studies. The rank probability showed that the best result for occlusoproximal cavities is the Hall technique (HALL) (1°). After that, the final ranking was NRCT (2°), CRT CMP (3°), CRT HV (4°), CRT RC (5°), ART (6°), CRT AM (7°) and UCT (8°) (Fig 5).

**Fig 6:**
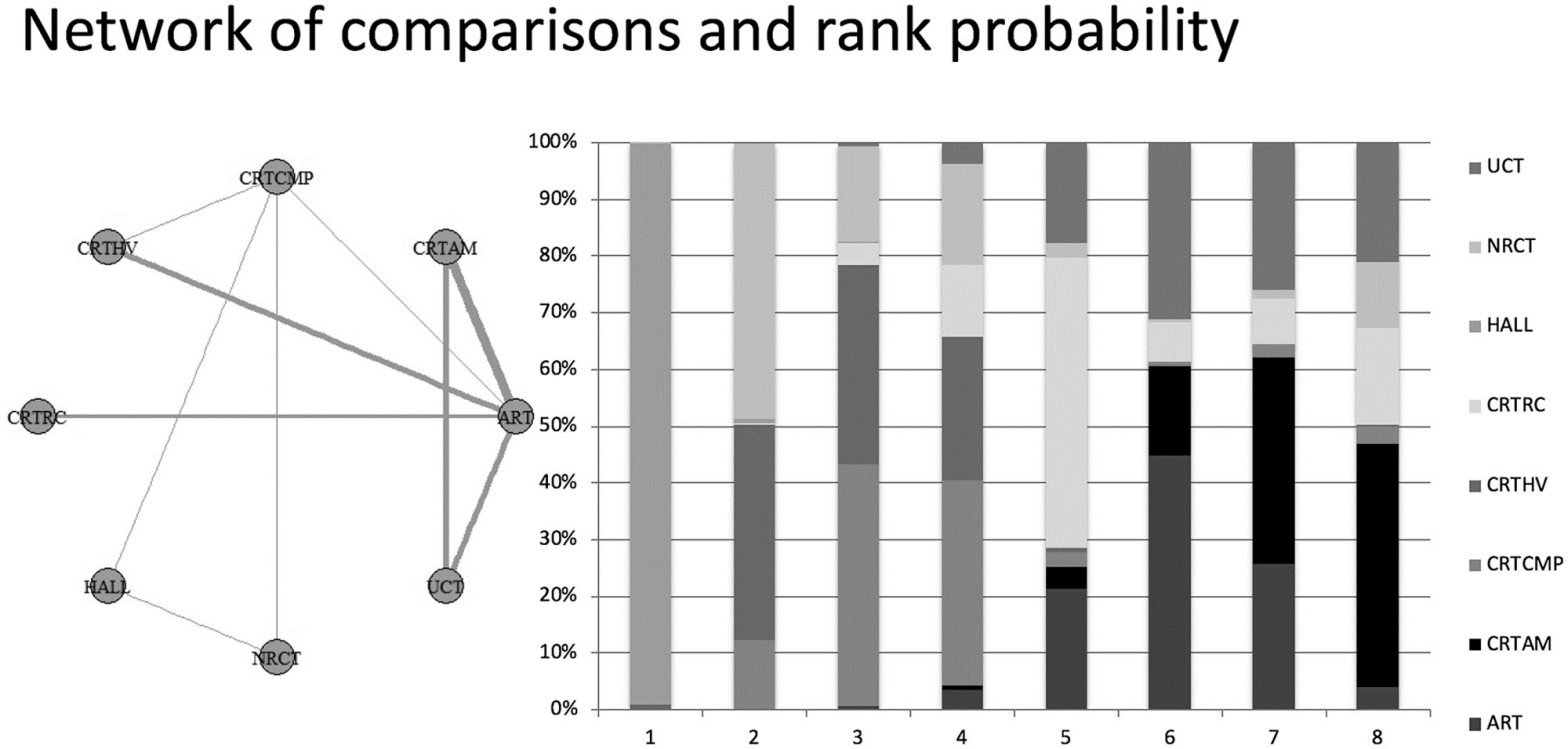
Network meta-analysis of the caries arrestment of different treatments options in dentin caries lesion on occlusal and smooth surfaces—Geometry of the network and probability ranking of the best behaviour of the treatments on occlusal surface and smooth surfaces.

Finally, three studies evaluated the caries arrestment assessment of the occlusal and smooth surfaces, considering five possible treatment options. Thus, a network meta-analysis was performed. The primary outcome of this comparison was caries arrestment (S5 Table). The results of the direct and indirect comparisons to provide the network meta-analysis was displayed in S5 and S6 Figs, respectively. Heterogeneity was not observed among the included studies. The rank probability showed that the best performance for this type of dentin caries lesion was two annual applications of silver diamine fluoride (SDF) (1°). After that, the final ranking was low-viscosity glass ionomer cement (LVGIC) (2°), one annual application of SDF (3°), three applications a year of SDF (4°), three applications a year of sodium fluoride (NaF) (5°) and interim restorative treatment (IRT) (6°) (Fig 6).

## Discussion

The lack of strong scientific support regarding treatments of dentin caries lesions in the primary teeth makes recommending the best available treatment challenging. Therefore, the present systematic review sought to verify the effect of the available treatments on caries lesion arrestment and the success rate for dentin caries lesions in the primary teeth, considering different depths of progression and the surface involved.

In general, we observed that the treatment efficacy to control caries lesions depends on the surfaces involved and the depth of progression. Two manuscripts considered only treatments for caries lesions on the outer half of the dentin of the occlusal surface, and a meta-analysis identified a similar performance with regard to caries arrestment for both treatment options (conventional restorative treatment with resin composite and sealing with resin sealant). This finding seems to occur in response to the blockade of the cavity from the biofilm formation by resin sealant; thus, even if the infected dentin has not been removed, there is no nutritional supply to the caries lesion progression [24,33]. However, when the success rate of a restorative treatment was considered as an outcome, CRT with resin composite demonstrated better performance than sealing with a resin sealant. Although this finding might result in a higher frequency of retreatments for sealed lesions [24], it is important to emphasize that the caries lesion arrestment should be considered as the primary outcome because it is the main goal of the restorative treatments. In this way, other advantages of the sealing technique can also be contemplated within pediatric dentistry as a less time-consuming restorative procedure that can positively affect the treatment of noncooperative children [24]. Alternatively, CRT can result in worse long-term prognoses for teeth because of the unnecessary removal of tooth substrate, even for a selective caries removal technique [34].

The results regarding caries lesions on the outer half of the dentin of the occlusal surface must be considered with caution because the inclusion criteria of these studies differed. Hesse et al. [20] considered a dentin cavity (ICDAS score = 5) with an entrance not wider than 3 mm in diameter in the enamel, whereas Borges et al. [32] selected noncavitated dentin caries lesions (ICDAS score = 4) for restoration. Although both studies included occlusal dentin caries lesions on the outer half of the dentin, the presence of a frank cavity in the dentin might lead to a high failure rate of the resin sealant because of its worse mechanical and bonding properties [35]. Furthermore, both studies revealed that the majority of the risk of bias assessment questions produced unclear results, demonstrating the necessity of additional well-designed clinical trials on this topic.

Likewise, without information about the depth of progression, the best results on the occlusal surfaces were verified for CRT with compomer, followed by ART. The difference between these techniques is specifically related to the use of rotary instruments and, in the current situation, the restorative material. Previous studies have observed better compomer mechanical properties that might explain this trend in performance [36,37]. Only one study evaluated this technique option with compomer, although the sample included approximately 1119 cavities, which might have affected the result of the network meta-analysis. Instead, this clinical trial had only one criterion show a low risk of bias, whereas five were rated as unclear. The uncertainty regarding potential bias should be an important factor for interpreting actual evidence. Conversely, five studies considered ART as a treatment option and appeared to provide greater scientific evidence, even though unclear information was also observed.

The network meta-analysis of the studies that treated occlusoproximal cavities resulted in a higher success rate when using the Hall technique. This phenomenon occurred because of the use of stainless-steel crowns, which can isolate the dentinal lesion from biofilm deposition and dietary challenges, leading to caries lesion arrestment [23]. Corroborating to this finding, the data from Innes et al. [32] demonstrated that this treatment option is more effective than CRT, with a success rate of 92% over an approximate five-year follow up. This study did not conduct a network meta-analysis since there was no isolated data according to the surfaces involved. This management strategy at least slows caries progression only via cavity sealing [32], which is linked to a possible additional benefit regarding the remineralization of caries lesions via the glass ionomer cement [38] used to repair the crowns [23]. This strategy might explain the positive performance on the occlusoproximal cavities.

Surprisingly, in opposition to this theory, the nonrestorative treatment was ranked as the second management possibility for occlusoproximal cavities. This technique enables biofilm removal via toothbrushing to arrest the caries lesion through the opening of the lesion with a high-speed bur and the removal of overhanging enamel [23]. Participants and parents or guardians receive dietary advice and specific toothbrushing instructions [23]. Moreover, unlike UCT, a fluoride varnish is applied to help the remineralization process [23]; this varnish is one of the reasons that we considered these techniques separately in this systematic review. Again, both techniques were evaluated by only one of the eight studies included in this analysis. The clinical trial that tested both treatments showed a high risk of bias on two items: blinding of the participants and examiners. This point must be weighted by the type of study design, which compared treatments that demand different approaches to cavity management, making the strategies of blinding challenging.

In relation to nonrestorative approaches to controlling caries on occlusal and smooth surfaces, two annual applications of 38% SDF showed a higher proportion of arresting active dentin caries lesions. The other silver diamine fluoride applications did not demonstrate similar performances. The positive effect of application every 6 months is in line with the recommended frequency of recall visits for children at high risk for caries [39,40]. Although the application of SDF can arrest caries lesions on occlusal and smooth surfaces, the authors claimed that lesions on buccal/lingual surfaces have a higher chance of becoming inactive using this technique based on biofilm accumulation, which is an important factor that negatively affects the progression of dentin caries lesions [26]. Of the three studies that evaluated these surfaces, only one tested two annual applications of SDF. Nevertheless, other authors affirmed that more applications act as an important component of its effectiveness [27], corroborating this hypothesis.

To obtain an overview of the effects of the treatments for dentin caries lesions in the primary teeth, different follow-up times were considered in the same analysis; this decision might be considered as a limitation of this systematic review. However, the small number of studies in this topic precludes subgroups analyses that consider the effect of follow-up time on the outcomes assessed. Importantly, the longevity of primary teeth is biologically brief. Thus, the follow-up time depends on the degree of tooth rhizolysis, which is correlated with patient age. The range of participants’ ages from the included studies is wide (3 to 10 years old); the longevity of the teeth and the survival rate of the treatments differ greatly across both situations.

The risk of bias analysis performed on the clinical trials showed that all studies received more unclear scores because of the uncertainty regarding potential bias in the questions, especially those related to allocation concealment, incomplete outcome data and baseline imbalances given that we were unable to identify this information. This finding shows that several authors fail to provide required methodological information even with guidelines for reporting clinical trials. On the other hand, because the observational study met the eligibility criteria, it was possible to include it in our systematic review. However, since the evaluated restorative techniques were not addressed in the others studies, the data from observational study were not included in the network meta-analyses. This study was designed as practice-based research and was evaluated by a tool for non-randomized studies of interventions. Most of the parameters were labeled as “yes”. Because of the low risk of bias in this study, we believe that this decision did not introduce an extra source of bias in our systematic review.

Another point to be highlighted is related to the evaluation of the quality of evidence, which showed low confidence in the results from the network meta-analysis regarding most of the analyses due to the risk of bias from the included studies. Furthermore, two studies included in the quantitative analysis presented greater than 30% dropout rates [18,28], which is the loss of participants threshold considered as acceptable in clinical trials. Although these data might jeopardize the final results because of the risk of bias and methodological failure, we opted to include them in the network meta-analysis to increase the sample size in the statistical analysis to provide more evidence regarding the chosen treatment for dentin caries lesions. Nevertheless, these parameters were addressed in the risk of bias analyses or reported as main characteristics of included studies in order to be considered with caution when interpreting the results. Additional well-designed studies that consider the points that affect the risk of bias of included studies and, specifically, the longevity of teeth as an outcome, should be conducted to guide pediatric dentistry in the management of dentin caries lesions supported by high-level and trustworthy evidence.

## Conclusions

The treatment of dentin caries lesions in primary teeth depends on the progression depth and surface involved. However, few studies exist, and most have a high risk of bias to provide enough evidence to strongly recommend the best treatment option.

## Supporting information

S1 Table PRISMA-NMA extension checklist. (DOCX)

S2 Table Eligible manuscripts excluded and the main reason to exclusion. (DOCX)

S3 Table MCT analysis for occlusal surface: Results from comparisons of the direct and indirect evidence as well as MTC evidence. (DOCX)

S4 Table MCT analysis for occlusoproximal surface: Results from comparisons of the direct and indirect evidence as well as MTC evidence. (DOCX)

S5 Table MCT analysis for occlusal and smooth surfaces: Results from comparisons of the direct and indirect evidence as well as MTC evidence. (DOCX)

S1 Fig Forest plots of pairwise comparisons from direct evidence of the network meta-analysis for occlusal surface. (DOCX)

S2 Fig Forest plots of pairwise comparisons from indirect evidence of the network meta-analysis for occlusal surface. (DOCX)

S3 Fig Forest plots of pairwise comparisons from direct evidence of the network meta-analysis for occlusoproximal surface. (DOCX)

S4 Fig Forest plots of pairwise comparisons from indirect evidence of the network meta-analysis for occlusoproximal surface. (DOCX)

S5 Fig Forest plots of pairwise comparisons from direct evidence of the network meta-analysis for occlusal and smooth surfaces. (DOCX)

S6 Fig Forest plots of pairwise comparisons from indirect evidence of the network meta-analysis for occlusal and smooth surface. (DOCX)
