# Supplementary material for: Scientific evidence for the management of dentin caries lesions in pediatric dentistry: A systematic review and network meta-analysis
Source: PLoS One. 2018 Nov 21;13(11):e0206296. doi: 10.1371/journal.pone.0206296 (PMC6248920; doi:10.1371/journal.pone.0206296)
Supplement: S2 Table — (DOCX) [file pone.0206296.s002.docx]

**S2 Table – Eligible manuscripts excluded and the main reason to exclusion.**

| Author/Year | Title | Main reason to exclusion |
| --- | --- | --- |
| Holst A, 1996 | A 3-year clinical evaluation of ketac-silver restorations in primary molars | Absence of a comparison group |
| Sharaf AA at al., 2004 | A clinical and radiographic evaluation of stainless steel crowns for primary molars | Absence of a comparison group |
| Deepa G et al., 2010 | A clinical evaluation of two glass ionomer cements in primary molars using atraumatic restorative treatment technique in India: 1 year follow up. | No evaluation of different restorative techniques |
| Stephenson J et al., 2010 | A competing risk survival analysis model to assess the efficacy of filling carious primary teeth. | Evaluation of other outcomes unrelated to this review |
| Yilmaz Y at al., 2006 | A one-year clinical evaluation of a high-viscosity glass ionomer cement in primary molars. | No evaluation of different restorative techniques |
| Bonifácio CC et al., 2013 | A preliminary clinical trial using flowable glass-ionomer cement as a liner in proximal-ART restorations: The operator effect | Absence of a comparison group |
| dos Santos MP et al., 2009 | A randomized trial of resin-based restorations in class I and class II beveled preparations in primary molars: 24-month results. | No evaluation of different restorative techniques |
| Mortada A et al., 2004 | A simplified technique for the restoration of severely mutilated primary anterior teeth | No evaluation of different restorative techniques |
| Barr-Agholme M et al., 1991 | A two-year clinical study of light-cured composite and amalgam restorations in primary molars. | Absence of a comparison group |
| de Amorim RG et al., 2014 | Amalgam and ART restorations in children: a controlled clinical trial. | More than one study was conducted in the same sample |
| Yee R, 2001 | An ART field study in western Nepal | No evaluation of different restorative techniques |
| Frencken JE et al., 1994 | An atraumatic restorative treatment (ART) technique: evaluation after one year. | No evaluation of different restorative techniques |
| Frencken JE et al., 1998 | ART restorations and glass ionomer sealants in Zimbabwe: Survival after 3 years | Absence of a comparison group |
| Beldüz Kara N et al., 2014 | Assessment of oral hygiene and periodontal health around posterior primary molars after their restoration with various crown types | Evaluation of other outcomes unrelated to this review |
| Abid A et al., 2002 | Atraumatic restorative treatment and glass ionomer sealants in Tunisian children: survival after 3 years. | No evaluation of different restorative techniques |
| Hesse D et al., 2016 | Bilayer technique and nano-filled coating increase success of approximal ART restorations: A randomized clinical trial | Absence of a comparison group |
| Prabhakar AR et al., 2015 | Cavity disinfection in minimally invasive dentistry-comparative evaluation of Aloe vera and propolis: A randomized clinical trial | No evaluation of different restorative techniques |
| Varpio M, 1993 | Changes in comprehensive dental care of the primary dentition from 1979 to 1989. II. Restorative treatment of primary molars in a population treated at a university dental clinic. | No evaluation of different restorative techniques |
| Ricci HA et al., 2010 | Chlorhexidine increases the longevity of in vivo resin-dentin bonds. | Evaluation of other outcomes unrelated to this review |
| Qvist V et al., 2004 | Class II restorations in primary teeth: 7-year study on three resin-modified glass ionomer cements and a compomer. | No evaluation of different restorative techniques |
| Attin T et al., 2000 | Class II restorations with a polyacid-modified composite resin in primary molars placed in a dental practice: results of a two-year clinical evaluation. | No evaluation of different restorative techniques |
| Fuks AB et al., 2000 | Clinical and radiographic assessment of Class II esthetic restorations in primary molars | No evaluation of different restorative techniques |
| Franzon R et al., 2007 | Clinical and radiographic evaluation of indirect pulp treatment in primary molars: 36 months follow-up. | No evaluation of different restorative techniques |
| Varpio M, 1993 | Clinical aspects of restorative treatment in the primary dentition. | No evaluation of different restorative techniques |
| Espelid I et al., 1999 | Clinical behaviour of glass ionomer restorations in primary teeth. | No evaluation of different restorative techniques |
| J Stoma, 2011 | Clinical comparison of two minimally invasive methods of caries treatment in deciduous teeth [Kliniczne porównanie dwóch minimalnie inwazyjnych metod leczenia próchnicy zebów mlecznych] | No evaluation of different restorative techniques |
| T Peric et al., 2009 | Clinical evaluation of a chemomechanical method for caries removal in children and adolescents | No evaluation of different restorative techniques |
| Akbay Oba A et al., 2009 | Clinical evaluation of a colored compomer in primary molars | No evaluation of different restorative techniques |
| J Rutar et al., 2000 | Clinical evaluation of a glass ionomer cement in primary molars. | Absence of a comparison group |
| Attin T et al., 1998 | Clinical evaluation of a hybrid composite and a polyacid-modified composite resin in Class-II restorations in deciduous molars. | Absence of a comparison group |
| Konde S et al., 2012 | Clinical evaluation of a new art material: Nanoparticulated resin-modified glass ionomer cement. | No evaluation of different restorative techniques |
| Papagiannoulis L et al., 1999 | Clinical evaluation of a polyacid-modified resin composite (compomer) in Class II restorations of primary teeth: A two-year follow-up study | No evaluation of different restorative techniques |
| Menezes JP et al., 2006 | Clinical evaluation of atraumatic restorations in primary molars: a comparison between 2 glass ionomer cements. | Absence of a comparison group |
| Kirzioglu Z et al., 2007 | Clinical evaluation of chemomechanical and mechanical caries removal: Status of the restorations at 3, 6, 9 and 12 months | No evaluation of different restorative techniques |
| FM Pascon et al., 2006 | Clinical evaluation of composite and compomer restorations in primary teeth: 24-month results. | No evaluation of different restorative techniques |
| M Peters et al., 1996 | Clinical evaluation of Dyract in primary molars: 1-year results. | No evaluation of different restorative techniques |
| Markovic D et al., 2008 | Clinical evaluation of glass-ionomer tunnel restorations in primary molars: 36 months results. | Absence of a comparison group |
| Welbury RR et al., 2000 | Clinical evaluation of paired compomer and glass ionomer restorations in primary molars: final results after 42 months. | Absence of a comparison group |
| L Granath et al., 1992 | Clinical evaluation of preventive and class-I composite resin restorations. | No evaluation of different restorative techniques |
| M.B. Sobhi et al., 2002 | Clinical evaluation of restorative materials in primary molars | Absence of a comparison group |
| Sengul F et al., 2015 | Clinical Evaluation of Restorative Materials in Primary Teeth Class II Lesions. | No evaluation of different restorative techniques |
| A. Fortuniak et al., 2012 | Clinical evaluation of the compomer restorations using different types of adhesive systems in the deciduous teeth - Annual and biennial observations | No evaluation of different restorative techniques |
| Grazyna marczuk-kolada et al., 2011 | Clinical evaluation of the effectiveness of atraumatic restorative treatment of caries in deciduous teeth - A three-year study | No evaluation of different restorative techniques |
| Phonghanyudh A et al., 2012 | Clinical evaluation of three caries removal approaches in primary teeth: a randomised controlled trial. | No evaluation of different restorative techniques |
| Yilmaz Y et al., 2004 | Clinical evaluation of two different methods of stainless steel esthetic crowns | No evaluation of different restorative techniques |
| Luo Y et al., 1999 | Clinical investigation of a high-strength glass ionomer restorative used with the ART technique in Wuhan, China: one-year results. | No evaluation of different restorative techniques |
| Lo EC et al., 2001 | Clinical investigation of two glass-ionomer restoratives used with the atraumatic restorative treatment approach in China: two-years results. | No evaluation of different restorative techniques |
| Mittal N et al., 2015 | Clinical outcomes of indirect composite restorations for grossly mutilated primary molars: a clinical observation. | No evaluation of different restorative techniques |
| Donly KJ et al., 1999 | Clinical performance and caries inhibition of resin-modified: Glass ionomer cement and amalgam restorations | Absence of a comparison group |
| Duggal MS et al., 2002 | Clinical performance of a compomer and amalgam for the interproximal restoration of primary molars: A 24-month evaluation | Evaluation of other outcomes unrelated to this review |
| Krämer N et al., 2001 | Clinical performance of a condensable metal-reinforced glass ionomer cement in primary molars | No evaluation of different restorative techniques |
| Faccin ES et al., 2009 | Clinical performance of art restorations in primary teeth: A survival analysis | Absence of a comparison group |
| Lorenna Bastos Lima Verde Nogueira et al., 2013 | Clinical performance of atraumatic restorative treatment in children with severe early childhood caries | Absence of a comparison group |
| Shah PV et al., 2004 | Clinical success and parental satisfaction with anterior preveneered primary stainless steel crowns | Absence of a comparison group |
| Schüler IM et al., 2014 | Clinical success of stainless steel crowns placed under general anaesthesia in primary molars: An observational follow up study | Absence of a comparison group |
| Ghaderi F et al., 2015 | Clinical Success Rate of Compomer and Amalgam Class II Restorations in First Primary Molars: A Two-year Study. | Absence of a comparison group |
| L Granath et al., 1992 | Clinical-evaluation of preventive and class-i composite resin restorations | No evaluation of different restorative techniques |
| Trairatvorakul C et al., 2004 | Comparative clinical evaluation of slot versus dovetail Class III composite restorations in primary anterior teeth | No evaluation of different restorative techniques |
| Yip HK et al., 2002 | Comparison of atraumatic restorative treatment and conventional cavity preparations for glass-ionomer restorations in primary molars: One-year results | More than one study was conducted in the same sample |
| Ertugrul F et al., 2010 | Comparison of conventional versus colored compomers for class II restorations in primary molars: a 12-month clinical study. | No evaluation of different restorative techniques |
| Gross LC et al., 2001 | Compomers as class II restorations in primary molars | No evaluation of different restorative techniques |
| Marks LA et al., 1999 | Conservative interproximal box-only polyacid modified composite restorations in primary molars, twelve-month clinical results. | No evaluation of different restorative techniques |
| Schwendicke F et al., 2016 | Conventional treatment, Hall Technique or immediate pulpotomy for carious primary molars: a cost-effectiveness analysis. | Evaluation of other outcomes unrelated to this review |
| Hübel S et al., 2003 | Conventional versus resin-modified glass-ionomer cement for Class II restorations in primary molars. A 3-year clinical study. | No evaluation of different restorative techniques |
| Maserejian NN et al., 2012 | Dental composite restorations and neuropsychological development in children: Treatment level analysis from a randomized clinical trial | Evaluation of other outcomes unrelated to this review |
| Trachtenberg F et al., 2009 | Does fluoride in compomers prevent future caries in children? | No evaluation of different restorative techniques |
| Andersson-Wenckert IE et al., 1997 | Durability of a polyacid-modified composite resin (compomer) in primary molars - A multicenter study | No evaluation of different restorative techniques |
| Marks LA et al., 1999 | Dyract versus Tytin Class II Restorations in Primary Molars: 36 Months Evaluation | Absence of a comparison group |
| Casagrande L et al., 2009 | Effect of adhesive restorations over incomplete dentin caries removal: 5-year follow-up study in primary teeth. | No evaluation of different restorative techniques |
| Leal SC et al., 2013 | Effect of different protocols for treating cavities in primary molars on the quality of life of children in Brazil - 1 year follow-up | Evaluation of other outcomes unrelated to this review |
| Kemoli AM et al., 2011 | Effects of oral hygiene, residual caries and cervical Marginal-gaps on the survival of proximal atraumatic restorative treatment approach restorations. | No evaluation of different restorative techniques |
| Qvist V et al., 2004 | Eight-year study on conventional glass ionomer and amalgam restorations in primary teeth. | No evaluation of different restorative techniques |
| Joly P et al., 2012 | Estimating survival of dental fillings on the basis of interval-censored data and multi-state models. | Evaluation of other outcomes unrelated to this review |
| Kotsanos N et al., 2011 | Evaluation of a resin modified glass ionomer serving both as indirect pulp therapy and as restorative material for primary molars. | Absence of a comparison group |
| Chibinski AC et al., 2013 | Evaluation of primary carious dentin after cavity sealing in deep lesions: a 10- to 13-month follow-up. | Evaluation of other outcomes unrelated to this review |
| Prabhakar AR et al., 2008 | Evaluation of the clinical behavior of resin modified glass ionomer cement on primary molars: a comparative one-year study. | No evaluation of different restorative techniques |
| Mijan MC et al., 2015 | Exfoliation rates of primary molars submitted to three treatment protocols after 3.5 years | Evaluation of other outcomes unrelated to this review |
| Casagrande L et al., 2008 | In vivo outcomes of indirect pulp treatment using a self-etching primer versus calcium hydroxide over the demineralized dentin in primary molars. | No evaluation of different restorative techniques |
| Casagrande L et al., 2010 | Indirect pulp treatment in primary teeth: 4-year results. | No evaluation of different restorative techniques |
| Falster CA et al., 2002 | Indirect pulp treatment: In vivo outcomes of an adhesive resin system vs calcium hydroxide for protection of the dentin-pulp complex | No evaluation of different restorative techniques |
| Kemoli AM et al., 2009 | Influence of the cavity-size on the survival rate of proximal ART restorations in primary molars. | No evaluation of different restorative techniques |
| Hilgert LA et al., 2014 | Is high-viscosity glass-ionomer-cement a successor to amalgam for treating primary molars? | More than one study was conducted in the same sample |
| Marks LA et al., 2000 | Ketac Molar Versus Dyract Class II restorations in primary molars: twelve month clinical results. | No evaluation of different restorative techniques |
| Kotsanos N et al., 2004 | Lack of effect of fluoride releasing resin modified glass ionomer restorations on the contacting surface of adjacent primary molars. a clinical prospective study. | Evaluation of other outcomes unrelated to this review |
| Qvist V et al., 1997 | Longevity and cariostatic effects of everyday conventional glass-ionomer and amalgam restorations in primary teeth: three-year results. | No evaluation of different restorative techniques |
| Pinto Gdos S et al., 2014 | Longevity of posterior restorations in primary teeth: results from a paediatric dental clinic. | No evaluation of different restorative techniques |
| Holan G et al., 1992 | Marginal leakage of impregnated Class 2 composites in primary molars: an in vivo study. | No evaluation of different restorative techniques |
| Singhal DK et al., 2016 | Microbiological analysis after complete or partial removal of carious dentin using two different techniques in primary teeth: A randomized clinical trial | Absence of a comparison group |
| Molina GF et al., 2014 | One year survival of ART and conventional restorations in patients with disability. | No evaluation of different restorative techniques |
| Yassen G, 2009 | One-year survival of occlusal ART restorations in primary molars placed with and without cavity conditioner. | No evaluation of different restorative techniques |
| Franzon R et al., 2014 | Outcomes of one-step incomplete and complete excavation in primary teeth: a 24-month randomized controlled trial. | Evaluation of other outcomes unrelated to this review |
| Foley J et al., 2004 | Partial caries removal and cariostatic materials in carious primary molar teeth: a randomised controlled clinical trial. | No evaluation of different restorative techniques |
| Nicolaisen S et al., 2000 | Performance of tunnel restorations at 3-6 years. | No evaluation of different restorative techniques |
| O'Connell AC et al., 2014 | Posterior preveneered stainless steel crowns: Clinical performance after three years | Absence of a comparison group |
| Rosenberg L et al., 2013 | Prospective study of indirect pulp treatment in primary molars using resin-modified glass ionomer and 2% chlorhexidine gluconate: A 12-month follow-up | No evaluation of different restorative techniques |
| Lo EC et al., 2001 | Provision of Atraumatic Restorative Treatment (ART) restorations to Chinese pre-school children - A 30-month evaluation | Absence of a comparison group |
| Varpio M et al., 1990 | Proximo-occlusal composite restorations in primary molars: Marginal adaptation, bacterial penetration, and pulpal reactions | Absence of a comparison group |
| Orhan AI et al., 2010 | Pulp exposure occurrence and outcomes after 1- or 2-visit indirect pulp therapy vs complete caries removal in primary and permanent molars | Absence of a comparison group |
| Alves dos Santos MP et al., 2010 | Randomised trial of resin-based restorations in Class I and Class II beveled preparations in primary molars: 48-month results. | No evaluation of different restorative techniques |
| Casagrande L et al., 2013 | Randomized clinical trial of adhesive restorations in primary molars. 18-month results. | No evaluation of different restorative techniques |
| Motta LJ et al., 2014 | Randomized controlled clinical trial of long-term chemo-mechanical caries removal using papacarie™ gel | No evaluation of different restorative techniques |
| Franzon R et al., 2015 | Randomized controlled clinical trial of the 24-months survival of composite resin restorations after one-step incomplete and complete excavation on primary teeth. | No evaluation of different restorative techniques |
| Qvist V et al., 2004 | Resin-modified and conventional glass ionomer restorations in primary teeth: 8-year results. | No evaluation of different restorative techniques |
| Folkesson UH et al., 1999 | Resin-modified glass ionomer cement restorations in primary molars. | No evaluation of different restorative techniques |
| Foley J et al., 2001 | Restoration of primary teeth: a study of copper phosphate cement. | No evaluation of different restorative techniques |
| Milgrom P, 2011 | Restorative treatment of primary teeth using compomer is not more effective than treatment with amalgam in preventing new tooth decay in children | Evaluation of other outcomes unrelated to this review |
| Kemoli AM et al., 2010 | Short communication: Influence of different isolation methods on the survival of proximal ART restorations in primary molars after two years. | No evaluation of different restorative techniques |
| Dutta BN et al., 2001 | Silver amalgam versus resin modified GIC class-II restorations in primary molars: twelve month clinical evaluation. | No evaluation of different restorative techniques |
| Oliveira CA et al., 2008 | Split mouth randomized controlled clinical trial of beveled cavity preparations in primary molars: an 18-Month follow up. | No evaluation of different restorative techniques |
| Mäkinen KK et al., 1995 | Stabilisation of rampant caries: polyol gums and arrest of dentine caries in two long-term cohort studies in young subjects. | No evaluation of different restorative techniques |
| Roshan NM et al., 2011 | Survival of occlusal ART restorations in primary molars placed in school environment and hospital dental setup-one year follow-up study | No evaluation of different restorative techniques |
| Bonifácio CC et al., 2013 | Survival rate of approximal-ART restorations using a two-layer technique for glass ionomer insertion | Absence of a comparison group |
| Hesse D et al., 2016 | Survival Rate of Atraumatic Restorative Treatment (ART) Restorations Using a Glass Ionomer Bilayer Technique with a Nanofilled Coating: A Bi-center Randomized Clinical Trial | No evaluation of different restorative techniques |
| Welbury RR et al., 1991 | The 5-year results of a clinical trial comparing a glass polyalkenoate (ionomer) cement restoration with an amalgam restoration. | Evaluation of other outcomes unrelated to this review |
| Luengas-Quintero E et al., 2013 | The atraumatic restorative treatment (ART) strategy in Mexico: two-years follow up of ART sealants and restorations. | No evaluation of different restorative techniques |
| Ersin NK et al., 2008 | The effect of a chlorhexidine containing cavity disinfectant on the clinical performance of high-viscosity glass-ionomer cement following ART: 24-month results. | Evaluation of other outcomes unrelated to this review |
| van Gemert-Schriks MC et al., 2008 | The effect of different dental treatment strategies on the oral health of children: A longitudinal randomised controlled trial | Absence of a comparison group |
| Bonifácio CC et al., 2013 | The effect of GIC-brand on the survival rate of proximal-ART restorations. | No evaluation of different restorative techniques |
| Seale NS, 2002 | The evaluation of stainless steel crowns, and their aesthetic modifications used for primary teeth restorations | No evaluation of different restorative techniques |
| Innes NP et al., 2007 | The Hall Technique; a randomized controlled clinical trial of a novel method of managing carious primary molars in general dental practice: acceptability of the technique and outcomes at 23 months. | Evaluation of other outcomes unrelated to this review |
| Soncini JA et al., 2007 | The longevity of amalgam versus compomer/composite restorations in posterior primary and permanent teeth: findings From the New England Children's Amalgam Trial. | No evaluation of different restorative techniques |
| N M Kilpatrick et al., 1995 | The use of a reinforced glass-ionomer cermet for the restoration of primary molars: a clinical trial. | No evaluation of different restorative techniques |
| Attin T et al., 2001 | Three-year follow up assessment of Class II restorations in primary molars with a polyacid-modified composite resin and a hybrid composite. | No evaluation of different restorative techniques |
| Santamaria R et al., 2014 | Trial shows partial caries removal is an effective technique in primary molars. | No evaluation of different restorative techniques |
| Hasselrot L, 1993 | Tunnel restorations. A 3 1/2-year follow up study of Class I and II tunnel restorations in permanent and primary teeth. | No evaluation of different restorative techniques |
| Carvalho TS et al., 2010 | Two years survival rate of Class II ART restorations in primary molars using two ways to avoid saliva contamination. | Absence of a comparison group |
| Topaloglu-Ak A et al., 2009 | Two years survival rate of class II composite resin restorations prepared by ART with and without a chemomechanical caries removal gel in primary molars. | Absence of a comparison group |
| aIbiyemi O et al., 2012 | Two years survival rate of occlusal ART restorations placed without tooth surface conditioning in a primary oral health care centre | Absence of a comparison group |
| MH Daou et al., 2009 | Two-Year Clinical Evaluation of Three Restorative Materials in Primary Molars | Absence of a comparison group |
| da Franca C et al., 2011 | Two-year evaluation of the atraumatic restorative treatment approach in primary molars class I and II restorations. | No evaluation of different restorative techniques |
| Kemoli AM et al., 2011 | Two-year survival rates of proximal atraumatic restorative treatment restorations in relation to glass ionomer cements and Postrestoration meals consumed. | Absence of a comparison group |
| Ferreira JM et al., 2013 | Use of glass ionomer cement containing antibiotics to seal off infected dentin: a randomized clinical trial. | No evaluation of different restorative techniques |
| Pendrys DG, 2010 | Use of the ART technique within the school setting can be a practical and effective method of treating caries among large populations of underserved children. | No evaluation of different restorative techniques |
| Croll TP et al., 2000 | Vitremer restorative cement for children: three clinicians' observations in three pediatric dental practices. | Absence of a comparison group |
| Hu X et al., 2013 | What happens to cavitated primary teeth over time? A 3.5-year prospective cohort study in China. | Evaluation of other outcomes unrelated to this review |
