# Supplementary material for: Scientific evidence for the management of dentin caries lesions in pediatric dentistry: A systematic review and network meta-analysis
Source: PLoS One. 2018 Nov 21;13(11):e0206296. doi: 10.1371/journal.pone.0206296 (PMC6248920; doi:10.1371/journal.pone.0206296)
Supplement: S3 Table — (DOCX) [file pone.0206296.s003.docx]

S3 Table – MCT analysis for occlusal surface: results from comparisons of the direct and indirect evidence as well as MTC evidence.

| COMPARISON | DIRECT EVIDENCE | INDIRECT EVIDENCE | MTC EVIDENCE | INCOSISTENCY |
| --- | --- | --- | --- | --- |
| ART x CRTAM | 1.0134 [0.9320; 1.1018] | 1.0 [0.92; 1.2] | 1.0 [0.87; 1.1] | 74.8% [42.9%; 88.9%] |
| ART x CRTCMP | 0.9796 [0.9329; 1.0286] | 1.0 [0.85; 1.3] | 0.97 [0.79; 1.2] | NA |
| ART x CRTHV | 1.0173 [0.9802; 1.0558] | 0.99 [0.89; 1.1] | 1.0 [0.89;1.1] | 0.0% [0.0%; 74.8%] |
| ART x CRTRC | 1.0102 [0.9878; 1.0332] | 1.0 [0.90; 1.2] | 0.99 [0.85; 1.1] | 39.2% [0.0%; 81.2%] |
| CRTAM x CRTHV | 1.0201 [0.9062; 1.1484] | 0.99 [0.85; 1.1] | 1.0 [0.89; 1.2] | 68.8% [0.0%; 90.9%] |
| CRTCMP x CRTHV | 0.9561 [0.9042; 1.0109] | 0.96 [0.79; 1.2] | 1.0 [0.84; 1.3] | NA |
| CRTAM x CRTCMP | NA | 1.0 [0.81; 1.3] | NA | NA |
| CRTAM x CRTRC | NA | 1.0 [0.84; 1.2] | NA | NA |
| CRTCMP x CRTRC | NA | 0.98 [0.78; 1.3] | NA | NA |

Abbreviations: ART: Atraumatic restorative treatment; CRT: Conventional restorative treatment; SSC: Stainless steel crown; NRCT: Nonrestorative caries treatment; UCT: Ultraconservative treatment; HT: Hall technique; IRT: Interim restorative treatment; AM: Amalgam; CMP: Compomer; HV: High-viscosity glass ionomer cement; RC: Resin composite.
