# Supplementary material for: Scientific evidence for the management of dentin caries lesions in pediatric dentistry: A systematic review and network meta-analysis
Source: PLoS One. 2018 Nov 21;13(11):e0206296. doi: 10.1371/journal.pone.0206296 (PMC6248920; doi:10.1371/journal.pone.0206296)
Supplement: S4 Table — (DOCX) [file pone.0206296.s004.docx]

S6 Table – MCT analysis for occlusoproximal surface: results from comparisons of the direct and indirect evidence as well as MTC evidence

| COMPARISON | DIRECT EVIDENCE | INDIRECT EVIDENCE | MTC EVIDENCE | INCOSISTENCY |
| --- | --- | --- | --- | --- |
| ART x CRTAM | 1.0134 [0.9752; 1.0531] | 0.99 [0.96; 1.0] | 1.0 [0.98;1.0] | 68.0% [32.9%; 84.8%] |
| ART x CRTCMP | 0.9268 [0.7977; 1.0769] | 1.1 [0.95; 1.3] | 0.89 [0.77;1.0] | NA |
| ART x CRTHV | 0.8619 [0.7880; 0.9426] | 1.2 [1.0; 1.3] | 0.86 [0.77;0.96] | 0.0% [0.0%; 76.8%] |
| ART x CRTRC | 0.9847 [0.9362; 1.0356] | 1.0 [0.95; 1.1] | 0.99 [0.93;1.0] | 0.0% [0.0%; 0.0%] |
| ART x UCT | 1.0318 [0.9645; 1.1038] | 0.99 [0.95; 1.0] | 1.0 [0.98;1.1] | 88.4% [75.6%; 94.5%] |
| CRTAM x UCT | 1.0183 [0.9648; 1.0747] | 1.0 [0.96; 1.0] | 1.0 [0.97;1.0] | 79.9% [52.7%; 91.5%] |
| CRTCMP x CRTHV | 0.9867 [0.8459; 1.1510] | 1.0 [0.90; 1.2] | 0.97 [0.82;1.1] | NA |
| CRTCMP x HALL | 0.7309 [0.6156; 0.8678] | NA | 0.74 [0.60;0.88] | NA |
| CRTCMP x NRCT | 0.9524 [0.7547; 1.2018] | 1.0 [0.81; 1.3] | 0.96 [0.76;1.2] | NA |
| HALL x NRCT | 1.303 [1.0999; 1.5436] | 1.5 [0.81; 1.3] | 1.3 [1.1;1.6] | NA |
| ART X HALL | NA | 1.5 [1.02; 2.0] | NA | NA |
| ART X NRCT | NA | 1.2 [0.86; 1.6] | NA | NA |
| CRTAM X CRTCMP | NA | 1.1 [0.96; 1.3] | NA | NA |
| CRTAM X CRTHV | NA | 1.2 [1.0; 1.3] | NA | NA |
| CRTAM X CRTRC | NA | 1.0 [0.95; 1.1] | NA | NA |
| CRTAM X HALL | NA | 1.5 [1.2; 2.0] | NA | NA |
| CRTAM X NRCT | NA | 1.2 [0.87; 1.6] | NA | NA |
| CRTCMP X CRTRC | NA | 0.90 [0.77; 1.1] | NA | NA |
| CRTCMP X UCT | NA | 0.88 [0.75; 1.0] | NA | NA |
| CRTHV X CRTRC | NA | 0.87 [0.77; 0.99] | NA | NA |
| CRTHV X HALL | NA | 1.3 [1.0; 1.7] | NA | NA |
| CRTHV X NRCT | NA | 1.0 [0.74; 1.3] | NA | NA |
| CRTHV X UCT | NA | 0.85 [0.75; 0.95] | NA | NA |
| CRTRC X HALL | NA | 1.5 [1.2; 1.9] | NA | NA |
| CRTRC X NRCT | NA | 1.1 [0.84; 1.6] | NA | NA |
| CRTRC X UCT | NA | 0.98 [0.91; 1.0] | NA | NA |
| UCT X NRCT | NA | 1.21 [0.87; 1.6] | NA | NA |

Abbreviations: ART: Atraumatic restorative treatment; CRT: Conventional restorative treatment; NRCT: Nonrestorative caries treatment; UCT: Ultraconservative treatment; HALL: Hall technique; HV: High-viscosity glass ionomer cement; RC: Resin composite; AM: Amalgam; CMP: Compomer; NA: Not applied
