# Supplementary material for: Scientific evidence for the management of dentin caries lesions in pediatric dentistry: A systematic review and network meta-analysis
Source: PLoS One. 2018 Nov 21;13(11):e0206296. doi: 10.1371/journal.pone.0206296 (PMC6248920; doi:10.1371/journal.pone.0206296)
Supplement: S5 Table — (DOCX) [file pone.0206296.s005.docx]

S9 Table – MCT analysis for occlusal and smooth surfaces: results from comparisons of the direct and indirect evidence as well as MTC evidence

| COMPARISON | DIRECT EVIDENCE | INDIRECT EVIDENCE | MTC EVIDENCE | INCOSISTENCY |
| --- | --- | --- | --- | --- |
| SDF 1 X SDF 2 | 0.87 [0.8; 0,95] | 1.1 [1.1; 1.3] | 0.3916 [0.2154; 0.7119] | 13% |
| SDF 1 X SDF 3 | 1.13 [0.95; 1.34] | 0.88 [0,74; 1.1] | 1.2148 [0.9242; 1.5967] | 13% |
| SDF 2 X SDF 3 | 1.0173 [0.9802; 1.0558] | 1.3 [1.1; 1.6] | 1.6860[0.955; 1.7967] | NA |
| NAF 3 X SDF 1 | 1.47 [1.22; 1.076] | 1.5 [1.2; 2.1] | 1.7703 [1.3527; 2.3168] | 13% |
| NAF 3 X SDF 3 | 1.30 [1.07; 1.57] | 1.3 [1.1; 1.6] | 1.4573 [1.1043; 1.9232] | NA |
| LVGIC X SDF 1 | 0.97 0.88; 1.07] | 1.7 [1.4; 2.2] | 0.8610 [0.5240; 1.4146] | 13% |
| LVGIC X SDF 2 | 1.11 [1.03; 1.2] | 1.1 [1.0; 2.2] | 2.1987 [1.2327; 3.9217] | NA |
| IRT X SDF 1 | 1.73 [1.38; 2.17] | 0.58 [0.46; 0.72] | 3.2149 [2.0362; 5.0758] | 13% |
| IRT X LVGIC | NA | 1.8 [1.4; 2.3] | NA | NA |
| IRT X NAF 3 | NA | 1.2 [0.89; 1.6] | NA | NA |
| IRT X SDF 2 | NA | 2.0 [1.6; 2.5] | NA | NA |
| IRT X SDF 3 | NA | 1.5 [1.2; 2.0] | NA | NA |
| LVGIC X NAF 3 | NA | 0.66 [0.54; 0.81] | NA | NA |
| LVGIC X SDF 3 | NA | 0.86 [0.70; 1.0] | NA | NA |
| NAF 3 X SDF 2 | NA | 1.7 [1.4; 2.1] | NA | NA |

Abbreviations: IRT: Interim restorative treatment; SDF: Silver diamine fluoride; LVGIC: Low-viscosity glass ionomer cement; NaF: Sodium fluoride; RS: resin sealant.
