# Supplementary material for: Scientific evidence for the management of dentin caries lesions in pediatric dentistry: A systematic review and network meta-analysis
Source: PLoS One. 2018 Nov 21;13(11):e0206296. doi: 10.1371/journal.pone.0206296 (PMC6248920; doi:10.1371/journal.pone.0206296)
Supplement: S1 Fig — (DOCX) [file pone.0206296.s006.docx]

S4 Figure – Forest plots of pairwise comparisons from direct evidence of the network meta-analysis for occlusal surface.

**
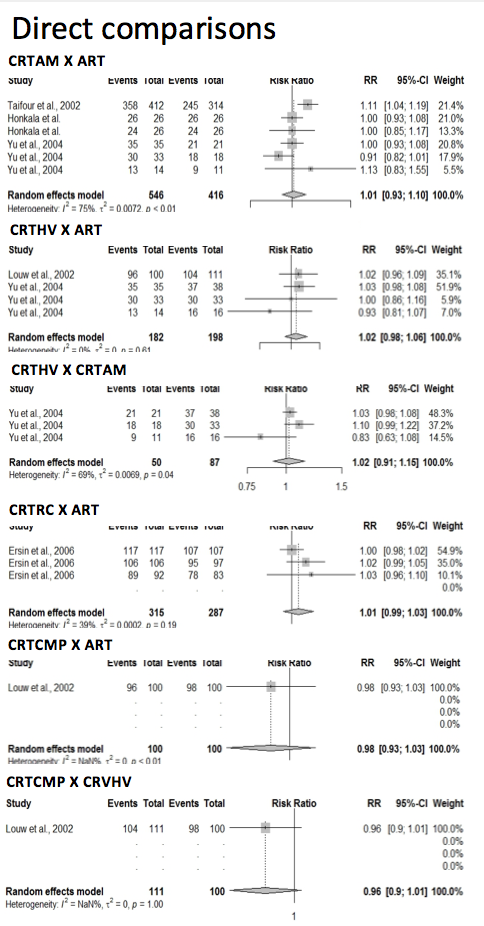
**

Abbreviations: ART: Atraumatic restorative treatment; CRT: Conventional restorative treatment; AM: Amalgam; CMP: Compomer; HV: High-viscosity glass ionomer cement; RC: Resin composite.
