# Supplementary material for: Scientific evidence for the management of dentin caries lesions in pediatric dentistry: A systematic review and network meta-analysis
Source: PLoS One. 2018 Nov 21;13(11):e0206296. doi: 10.1371/journal.pone.0206296 (PMC6248920; doi:10.1371/journal.pone.0206296)
Supplement: S6 Fig — (DOCX) [file pone.0206296.s011.docx]

S11 Figure – Forest plots of pairwise comparisons from indirect evidence of the network meta-analysis for occlusal and smooth surface.

**
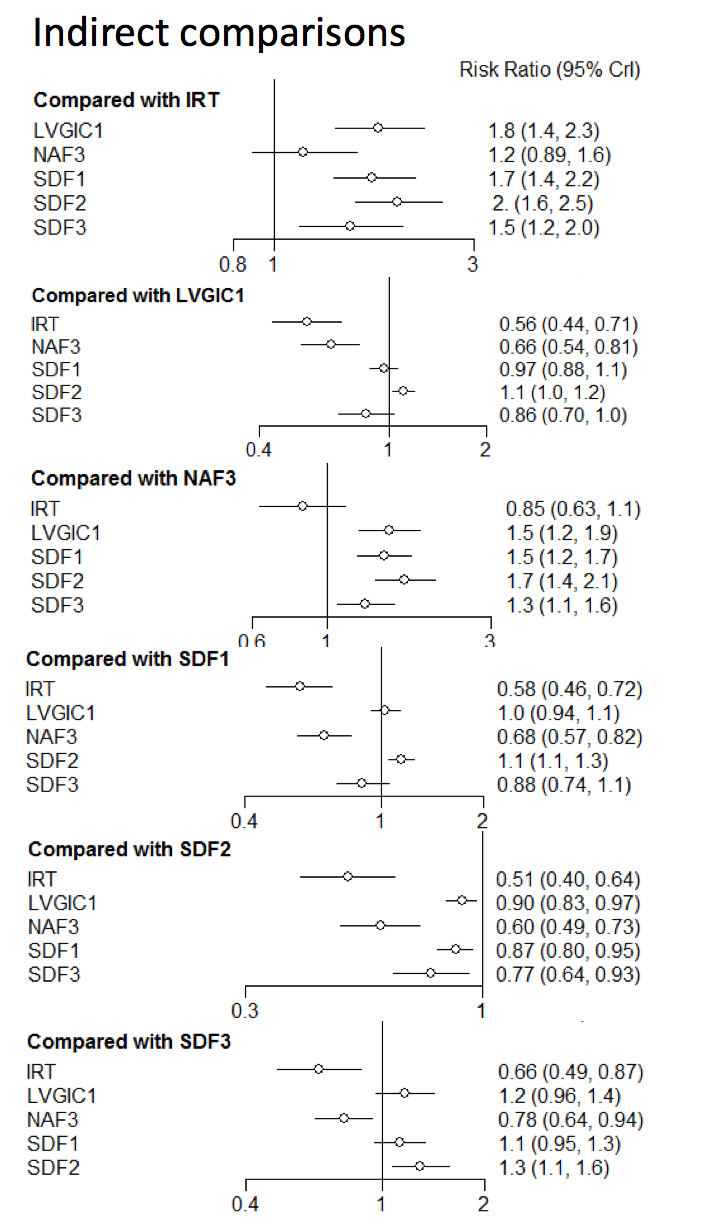
**

Abbreviations: IRT: Interim restorative treatment; SDF: Silver diamine fluoride; LVGIC: Low-viscosity glass ionomer cement; NaF: Sodium fluoride.
